# Radium-223 in combination with paclitaxel in cancer patients with bone metastases: safety results from an open-label, multicenter phase Ib study

**DOI:** 10.1007/s00259-018-4234-6

**Published:** 2018-12-13

**Authors:** Ravit Geva, Juanita Lopez, Sarah Danson, Heikki Joensuu, Avivit Peer, Samuel J. Harris, Fabricio Souza, Kaline M. C. Pereira, Ruth Perets

**Affiliations:** 1 Division of Oncology, Tel Aviv Sourasky Medical Center, Tel-Aviv University, 6 Weizmann Street, 64239 Tel Aviv, Israel; 2 Drug Development Unit, The Royal Marsden NHS Foundation Trust and Institute of Cancer Research, 15 Cotswold Road, Sutton, London, SM2 5NG UK; 3 Department of Oncology and Metabolism, Sheffield Experimental Cancer Medicine Centre, University of Sheffield and Sheffield Teaching Hospitals NHS Foundation Trust, Whitham Road, Sheffield, S10 2SJ UK; 4 Department of Oncology, Helsinki University Hospital and University of Helsinki, Haartmaninkatu 4, 00029 Helsinki, Finland; 5 Oncology Division, Rambam Health Care Campus, 8 Haaliya Street, Haifa, Israel; 6 Department of Medical Oncology, The Royal Marsden NHS Foundation Trust and Institute of Cancer Research, 15 Cotswold Road, Sutton, London, SM2 5NG UK; 7 Clinical Pharmacology Leader Oncology, Bayer HealthCare Pharmaceuticals, 100 Bayer Boulevard, Whippany, NJ USA; 8 Medical and Data Management, Bayer HealthCare Pharmaceuticals, 100 Bayer Boulevard, Whippany, NJ USA; 9 Oncology Division, Rambam Health Care Campus, 8 Ha’Aliya Hashniya, and Technion – Israel Institute of Technology, 1 Efron Street, Haifa, Israel

**Keywords:** Bone metastases, Cancer, Paclitaxel, Radium-223 dichloride, Safety

## Abstract

**Purpose:**

Concomitant treatment with radium-223 and paclitaxel is a potential option for cancer patients with bone metastases; however, myelosuppression risk during coadministration is unknown. This phase Ib study in cancer patients with bone metastases evaluated the safety of radium-223 and paclitaxel.

**Methods:**

Eligible patients had solid tumor malignancies with ≥2 bone metastases and were candidates for paclitaxel. Treatment included seven paclitaxel cycles (90 mg/m^2^ per week intravenously per local standard of care; 3 weeks on/1 week off) plus six radium-223 cycles (55 kBq/kg intravenously; one injection every 4 weeks, starting at paclitaxel cycle 2). The primary end point was percentage of patients with grade 3/4 neutropenia or thrombocytopenia during coadministration of radium-223 and paclitaxel (cycles 2, 3) versus paclitaxel alone (cycle 1).

**Results:**

Of 22 enrolled patients, 15 were treated (safety population), with 7 completing all six radium-223 cycles. Treated patients had primary cancers of breast (*n* = 7), prostate (*n* = 4), bladder (*n* = 1), non–small cell lung (*n* = 1), myxofibrosarcoma (*n* = 1), and neuroendocrine (*n* = 1). No patients discontinued treatment from toxicity of the combination. In the 13 patients who completed cycle 3, the rates of grade 3 neutropenia in cycles 2 and 3 were 31% and 8%, respectively, versus 23% in cycle 1; there were no cases of grade 4 neutropenia or grade 3/4 thrombocytopenia. Breast cancer subgroup safety results were similar to the overall safety population.

**Conclusion:**

Radium-223 was tolerated when combined with weekly paclitaxel, with no clinically relevant additive toxicities. This combination should be explored further in patients with bone metastases.

**Electronic supplementary material:**

The online version of this article (10.1007/s00259-018-4234-6) contains supplementary material, which is available to authorized users.

## Introduction

Patients with cancer who develop bone metastases face additional challenges that include increased incidence of skeletal-related events, reduced quality of life, and increased economic burden of the disease [1–5]. Combination treatments that utilize complementary mechanisms of action and non-overlapping toxicity profiles are a rational choice for these patients, and there is a clinical need to identify safe and effective combinations that treat both systemic and metastatic disease.

Radium-223 dichloride (radium-223) is a targeted alpha emitter that mimics calcium and selectively binds to areas of active bone remodeling [6–8]. It emits high-energy, short-range (<100 μm) alpha particles that lead to double-stranded DNA breaks in nearby cells. Prostate cancer preclinical models have shown that radium-223 may act via a dual mechanism of action through cytotoxic effects on tumor cells and stabilization of the bone microenvironment [9]. Similar findings were observed in breast cancer preclinical models, suggesting efficacy regardless of primary tumor origin [10].

In the phase III ALSYMPCA trial, radium-223 significantly prolonged overall survival (OS) and delayed time to first symptomatic skeletal event (SSE) when compared with placebo, and was well tolerated with low rates of myelosuppression in metastatic castration-resistant prostate cancer (CRPC) patients [11]. These results led to radium-223 approval for the treatment of CRPC patients with symptomatic bone metastases and no known visceral metastatic disease [12]. In a randomized phase I/IIa study of metastatic CRPC patients, radium-223 was administered in combination with the taxane chemotherapy docetaxel [13]. Compared with patients who received docetaxel alone, patients co-treated with radium-223 plus docetaxel had less grade 3/4 treatment-emergent adverse events (AEs), longer progression-free survival (PFS), and longer time to radiographic or clinical progression [13]. Based on the observed data, a myelosuppression model was designed.

Combining radium-223 with chemotherapy may broaden treatment options for patients with malignancies treated with taxane chemotherapy. Instead of testing each drug combination in a clinical trial setting, developing a model that predicts myelosuppression may provide useful data to better understand the myelosuppressive potential of novel drug combinations. Myelosuppression modeling data from a drug with a mechanism of action similar to that of docetaxel could be used to support the tool’s validation. For this reason, paclitaxel was chosen for use in this radium-223 combination study.

Paclitaxel is a taxane chemotherapy that is in the same class as docetaxel [14]. It exhibits antitumor activity by binding tubulin and stabilizing nonfunctional microtubule bundles to block normal mitotic spindle development and subsequent cell division [15]. Neutropenia is commonly associated with paclitaxel [16]; however, studies have shown that paclitaxel had less hematologic toxicity when administered weekly versus every 3 weeks [14].

Concomitant treatment with radium-223 and paclitaxel is a possible treatment strategy for cancer patients with bone metastases. Radium-223 and paclitaxel have potentially synergistic mechanisms of action; however, both agents impact hematologic parameters, and myelosuppression risk during coadministration is unknown. As it is in the same class as docetaxel [14], paclitaxel administered in combination with radium-223 is expected to have a similar tolerability profile as in the randomized phase I/IIa radium-223 plus docetaxel study [13]. Paclitaxel has a generally moderate effect on neutrophil counts [14], allowing better assessment of the potentially additive effect of radium-223 when both are used in combination. It is clinically relevant to assess overall safety and potential hematologic toxicity during coadministration of radium-223 and paclitaxel, as this may be a potentially new combination treatment strategy for patients with bone metastases. This open-label, multicenter, nonrandomized phase Ib study evaluated the safety and tolerability of concomitant treatment with radium-223 and paclitaxel in cancer patients with bone metastases. To the best of our knowledge, this study is the first to evaluate concomitant use of radium-223 and paclitaxel.

## Patients and methods

### Patient population

Eligible patients were male or female aged ≥18 years, had a confirmed malignant solid tumor with at least two bone metastases, and were eligible for treatment with paclitaxel as a single agent. Visceral metastases were allowed. Documentation of premenopausal or postmenopausal status was required for female patients; postmenopausal status was defined either by ≥1 year of amenorrhea in the absence of other biologic or physiologic causes or by surgical menopause with bilateral oophorectomy. Premenopausal patients were required to have a serum pregnancy test within 7 days before starting study treatment. Male and female patients of reproductive potential were required to agree to using two acceptable methods of contraception simultaneously from the time of signing the informed consent form to 6 months after the last radium-223 injection. Additional inclusion criteria included life expectancy of ≥16 weeks, Eastern Cooperative Oncology Group (ECOG) performance status of 0 or 1, and adequate bone marrow, liver, kidney, and blood clotting function.

Patients were excluded if they had prior systemic therapy with radionuclides; imminent or established spinal cord compression; active brain metastases or meningeal tumors; prior hemibody external radiotherapy; major surgical procedure, open biopsy, or significant traumatic injury within 28 days before starting study treatment; bone fracture in weight-bearing bones without acceptable orthopedic stabilization within 4 weeks prior to starting study treatment; confirmed Paget’s disease of the bone; uncontrolled seizure disorder requiring anticonvulsant medication; severe cardiac conduction disorders requiring antiarrhythmic therapy (except for beta-blockers); pleural effusion or ascites causing respiratory compromise; ongoing interstitial lung disease; or evidence of peripheral neuropathy that was greater than grade 1.

Additional exclusion criteria included arterial or venous thrombotic or embolic events within 6 months before starting study treatment; deep vein thrombosis within 3 months before starting study treatment (except for adequately treated catheter-related venous thrombosis occurring ≥1 month before start of study medication); evidence or history of bleeding diathesis; unresolved toxicity higher than grade 1 attributed to any prior therapy or procedure (excluding alopecia or anemia with hemoglobin ≥9 mg/dL); blood transfusion or use of erythropoietin within 6 weeks prior to start of study treatment; platelet transfusions within 3 weeks prior to start of study treatment; use of biologic response modifiers (e.g., granulocyte-macrophage colony-stimulating factor [GM-CSF] or granulocyte colony-stimulating factor [G-CSF]) within 6 weeks prior to start of study treatment; intake of clozapine within 4 weeks before start of study treatment; or history of organ allograft, cardiac disease, Crohn’s disease or ulcerative colitis, bone marrow dysplasia, human immunodeficiency virus, or active or chronic hepatitis B or C virus requiring antiviral treatment. All patients provided written informed consent.

### Study design and treatment

**Fig. 1:**
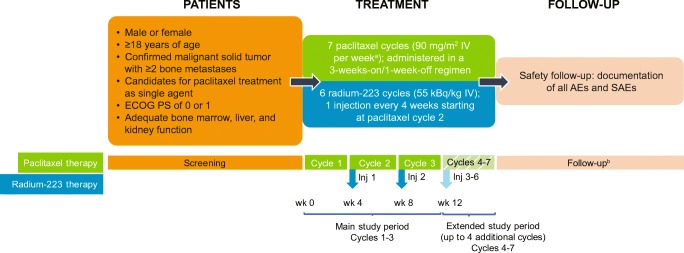
Study design. In this phase Ib study, eligible patients were to receive one cycle of paclitaxel alone, followed by up to six cycles of radium-223 in combination with paclitaxel. ^a^Per local standard of care. ^b^Safety follow-up started 30 days after last study drug administration. *AEs* adverse events, *ECOG PS* Eastern Cooperative Oncology Group performance status, *Inj* injection, *SAEs* serious adverse events

This open-label, multicenter, nonrandomized phase Ib study (NCT02442063) was designed to evaluate the safety and tolerability of radium-223 administered in combination with paclitaxel in cancer patients with bone metastases. Treatment included up to seven paclitaxel cycles (90 mg/m^2^ IV per week as per local standard of care; administered in a 3 weeks on/1 week off regimen, starting in cycle 1) combined with up to six radium-223 cycles (55 kBq/kg IV; one injection every 4 weeks, starting at paclitaxel cycle 2; Fig. 1). All patients were premedicated with corticosteroids and antihistamines (H1 and H2 antagonists) before each paclitaxel infusion, according to local standard of care. Radium-223 was administered as a slow injection after the patient had received paclitaxel. The primary end point was percentage of patients with grade 3/4 neutropenia or thrombocytopenia during treatment with radium-223 plus paclitaxel (cycles 2 and 3) versus paclitaxel alone (cycle 1).

Paclitaxel dose reductions and dose delays or interruptions were permitted, to account for individual patient tolerance. Neutrophil and platelet counts were required to be ≥1.5 × 10^9^/L and ≥ 100 × 10^9^/L, respectively, before each paclitaxel administration. Paclitaxel doses scheduled for days 8 and 15 were permitted to be delayed by up to 2 days; any delay longer than 2 days was considered a missed dose that was not replaced. Radium-223 dose reductions were not permitted. Radium-223 doses could be delayed for up to 4 weeks (maximum 8 weeks between two injections) for recovery of AEs. If administration was delayed for >4 weeks, radium-223 was discontinued and the patient entered follow-up.

The main study observation period was the first 12 weeks (i.e., cycles 1–3), which included one cycle of paclitaxel administered alone followed by two cycles of radium-223 in combination with paclitaxel. A 30-day follow-up was planned for each patient after completion of the last cycle of study treatment (i.e., final radium-223 injection in cycle 7). The planned duration of treatment was approximately 32 weeks (i.e., one cycle of paclitaxel alone plus six cycles of paclitaxel in combination with radium-223 at 4-week intervals plus 30 days follow-up).

The review boards at all participating centers reviewed and approved the study protocol. The study was conducted in accordance with the Declaration of Helsinki and the International Conference on Harmonisation guidelines for Good Clinical Practice.

### Assessments

Safety was monitored and evaluated throughout the study and 30-day follow-up period. Safety assessments included AEs and laboratory parameters (e.g., blood tests). AEs were analyzed using the National Cancer Institute’s Common Terminology Criteria for AEs, version 4.03 (NCI CTCAE v4.03); separate AE tables were also generated using terminology from the Medical Dictionary for Regulatory Activities (MedDRA) version 19.1. Blood samples to determine platelet and neutrophil counts for the primary end point were collected twice weekly (i.e., every 3–4 days) during cycles 1–3. Thereafter, blood samples were taken every 2 weeks until 4 weeks after the last radium-223 injection. Analysis of hematologic parameters was performed at local laboratories.

### Statistical analysis

Given the exploratory nature of this study, no formal sample size calculation was performed. All patients who received at least one dose of study drug were included in the safety population. A descriptive subgroup analysis of patients with breast cancer was performed and compared with the safety population.

All parameters were summarized using descriptive statistics. Parameters were summarized by treatment and cycle, if applicable, using SAS version 9.2 or higher. All planned analyses were explorative, and a confirmatory statistical analysis was not intended.

## Results

### Patients

The study was conducted at five centers in Finland, Israel, and the United Kingdom from August 18, 2015 to October 24, 2016. Of the 22 patients enrolled, 15 received at least one dose of paclitaxel and were included in the safety population (Supplementary Table S1). Patient demographics and baseline clinical characteristics are shown in Table 1. Breast cancer was the most common tumor type, present in 7 (47%) of 15 patients (breast cancer subgroup). Compared with the safety population, there were fewer patients in the breast cancer subgroup with prior taxane therapy (29% vs. 53%), but rates of ≥3 prior chemotherapy regimens were similar (43% vs. 47%).

**Table 1:** Patient demographics and baseline clinical characteristics

Characteristics	Safety population *n* = 15	Breast cancer subgroup *n* = 7
Age, median (range), years	61 (45–76)	58 (45–68)
ECOG PS, *n* (%)		
0	6 (40)	5 (71)
1	8 (53)	2 (29)
Progressive disease at study entry, *n* (%)	15 (100)	7 (100)
Status of primary tumor, *n* (%)		
R0, complete tumor resection with all margins histologically negative	8 (53)	5 (71)
Resected, status of residual tumor unknown	4 (27)	0
Unresected	3 (20)	2 (29)
Breast cancer tumors, *n* (%)	7 (47)	7 (100)
HER2-	7 (100)	7 (100)
Hormone receptor status		
ER+/PR+	4 (57)	4 (57)
ER+/PR-	1 (14)	1 (14)
ER+/PR unknown	1 (14)	1 (14)
ER-/PR-	1 (14)	1 (14)
Other tumor types, *n* (%)		
Prostate cancer	4 (27)	0
Bladder cancer	1 (7)	0
Non–small cell lung cancer	1 (7)	0
Neuroendocrine cancer	1 (7)	0
Myxofibrosarcoma	1 (7)	0
Number of bone metastases at baseline, *n* (%)		
2	1 (7)	1 (14)
3	3 (20)	1 (14)
4	4 (27)	3 (43)
5	2 (13)	1 (14)
9	3 (20)	0
10	2 (13)	1 (14)
Number of prior chemotherapy regimens, *n* (%)		
0	3 (20)	2 (29)
1	1 (7)	0
2	4 (27)	2 (29)
≥ 3	7 (47)	3 (43)

*BC* Breast cancer, *ECOG PS* Eastern Cooperative Oncology Group performance status, *ER* Estrogen receptor, *HER2* Human epidermal growth factor receptor 2, *PR* Progesterone receptor, *+* Positive, − Negative

All 15 (100%) patients received at least one paclitaxel injection. The median number of paclitaxel cycles received was 6 (range, 1–7 cycles), with a median treatment time of 22 weeks (range, 2–30 weeks). A total of 14 (93%) of 15 patients received treatment with radium-223; 1 patient was withdrawn from the study during cycle 1 and did not receive a radium-223 dose. The median number of radium-223 injections received was 5.5 (range, 1–6 injections). A total of 7 (47%) of 15 patients completed treatment with six radium-223 injections and seven paclitaxel infusions.

Eight (53%) of 15 patients discontinued study treatment. The primary reasons for treatment discontinuation were radiologic disease progression (27%), AEs associated with disease progression (7%), AEs not associated with disease progression (13%), and patient withdrawal (7%). No patients discontinued treatment because of toxicity from the treatment combination.

Breast cancer patients, versus the safety population, had a slightly longer median treatment duration for radium-223 (6 vs. 5.5 cycles) and paclitaxel (7 vs. 6 cycles), and more patients who completed six radium-223 doses (57% vs. 47%). Treatment discontinuation related to disease progression occurred in 29% of breast cancer patients versus 33% in the safety population.

### Primary end point

**Fig. 2:**
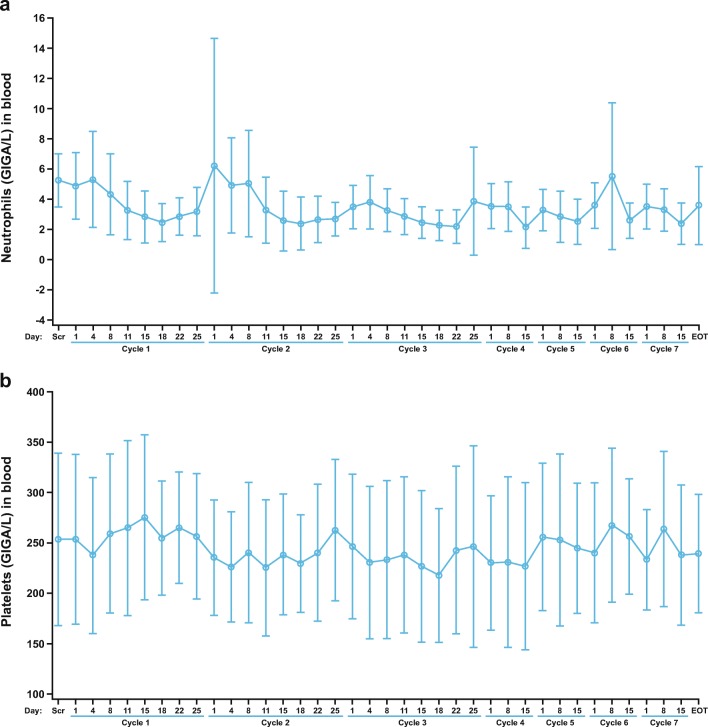
Hematology values over time (means and standard deviations) for neutrophils (a) and platelets (b) in the safety population, from screening to end of treatment. *EOT* End of treatment, *Scr* Screening

In the safety population, 13 (87%) of 15 patients completed treatment cycles 1–3 and were included in the analysis of the primary end point. In these patients, grade 3 neutropenia rates were 31% in cycle 2 and 8% in cycle 3, versus 23% in cycle 1; there were no cases of grade 4 neutropenia or grade 3/4 thrombocytopenia during cycles 1–3 (Table 2). In the breast cancer subgroup, all seven patients completed treatment cycles 1–3. In these patients, grade 3 neutropenia rates were 43% in cycle 2 and 14% in cycle 3, versus 29% in cycle 1; there were no cases of grade 4 neutropenia or grade 3/4 thrombocytopenia during cycles 1–3 (Table 2). The overall number of patients with grade 3/4 neutropenia or thrombocytopenia during the study (cycles 1–7) is shown by treatment cycle in Supplementary Table S2. Neutrophil and platelet values from screening to end of treatment for the safety population are shown in Fig. 2. Mean neutrophil levels did not exceed grade 2 during treatment and tended to recover at the end of each cycle; however, postbaseline mean neutrophil counts did not return to pre-dose levels for the majority of timepoints (Fig. 2 A). Overall, there did not appear to be a trend toward declining neutrophil levels over time, even after six cycles of combination treatment. The peaks in mean neutrophil counts observed at cycle 2, day 8 and cycle 6, day 8 were due to G-CSF, which was permitted during the study when clinically indicated or at the discretion of the Investigator. Only one patient took G-CSF for myelosuppression. Postbaseline mean platelet counts returned to baseline values at several timepoints (Fig. 2 B).

**Table 2:** Grade 3 or 4 neutropenia or thrombocytopenia in patients who completed treatment cycles 1–3 (primary endpoint)

Population, cycle	Patients, *n*	Neutropenia, *n* (%)		Thrombocytopenia, *n* (%)	
		Grade 3	Grade 4	Grade 3	Grade 4
Safety population					
Cycle 1	13	3 (23)	0	0	0
Cycle 2	13	4 (31)	0	0	0
Cycle 3	13	1 (8)	0	0	0
Breast cancer subgroup					
Cycle 1	7	2 (29)	0	0	0
Cycle 2	7	3 (43)	0	0	0
Cycle 3	7	1 (14)	0	0	0

The lower grade 3 neutropenia rate observed in cycle 3 versus cycle 2 was likely driven by paclitaxel dose modifications (either dose interruptions or dose reductions) or G-CSF use. Overall, 13 (87%) of 15 patients had paclitaxel dose modifications (interruptions or delays in 11 [73%] and reductions in 5 [33%]); 5 (33%) patients had radium-223 dose modifications (all interruptions or delays).

### Safety

All 15 (100%) patients in the safety population experienced at least one treatment-emergent AE (TEAE), and also reported at least one paclitaxel-related TEAE as determined by the investigator (Table 3). A total of 10 (67%) of 15 patients experienced a radium-223–related TEAE as determined by the investigator.

**Table 3:** Treatment-emergent adverse events

Patients with TEAEs, *n* (%)	Safety population, *n* = 15	Breast cancer subgroup, *n* = 7
Any TEAE	15 (100)	7 (100)
Grade 3 or 4	9 (60)	2 (29)
Grade 5^a^	2 (13)	2 (29)
Serious	6 (40)	2 (29)
Leading to permanent discontinuation of study drug	2 (13)	1 (14)
Any paclitaxel-related TEAE^b^	15 (100)	7 (100)
Grade 3 or 4	8 (53)	3 (43)
Serious	2 (13)	0
Leading to permanent discontinuation of study drug	1 (7)	0
Any radium-223–related TEAE^b^	10 (67)	5 (71)
Grade 3 or 4	4 (27)	1 (14)
Serious	1 (7)	0
Leading to permanent discontinuation of study drug	0	0

^a^Both associated with disease progression and unrelated to study treatment

^b^As determined by the investigator

*TEAEs* Treatment-emergent adverse events

TEAEs occurring in >15% of patients are shown in Table 4. Neutropenia was the most common grade 3/4 TEAE; it occurred in 6 (40%) of 15 patients in the safety population and 3 (43%) of 7 patients in the breast cancer subgroup. Three patients experienced a fracture: one patient had a pathologic fracture (proximal right humerus) that was considered not serious, one patient had a pathologic L3 fracture that was considered not serious, and one patient had a non-pathologic right hip fracture of an unspecified section of the neck of the femur (femoral neck hip fracture) due to a fall that was considered a serious AE (SAE) and led to hospitalization; this patient then experienced sepsis and died. This patient received only one radium-223 injection, and the fracture occurred 42 days after the last (only) radium-223 dose. None of the observed fractures were considered related to study drug by the investigator.

**Table 4:** Treatment-emergent adverse events in >15% of patients during cycles 1–7

Patients with TEAEs, *n* (%)^a^	Safety population, *n* = 15			Breast cancer subgroup^b^ , *n* = 7		
	All grades	Grade 3	Grade 4	All Grades	Grade 3	Grade 4
Hematologic AEs						
Anemia	4 (27)	1 (7)	0	3 (43)	1 (14)	0
Leukopenia	3 (20)	2 (13)	0	3 (43)	2 (29)	0
Neutropenia	9 (60)	4 (27)	2 (13)	5 (71)	2 (29)	1 (14)
Nonhematologic AEs						
Vision blurred	3 (20)	0	0	1 (14)	0	0
Abdominal pain	5 (33)	1 (7)	0	4 (57)	1 (14)	0
Constipation	4 (27)	1 (7)	0	3 (43)	1 (14)	0
Diarrhea	8 (53)	0	0	4 (57)	0	0
Nausea	5 (33)	0	0	4 (57)	0	0
Vomiting	6 (40)	0	0	5 (71)	0	0
Fatigue	9 (60)	1 (7)	0	5 (71)	0	0
Peripheral edema	5 (33)	0	0	2 (29)	0	0
Urinary tract infection	3 (20)	0	0	1 (14)	0	0
Pain in extremity	3 (20)	1 (7)	0	1 (14)	0	0
Peripheral neuropathy	9 (60)	0	0	6 (86)	0	0
Alopecia	10 (67)	0	0	6 (86)	0	0
Rash	4 (27)	0	0	1 (14)	0	0

^a^According to Medical Dictionary for Regulatory Activities (MedDRA) preferred term. Breast cancer subgroup according to CTCAE terminology: leukopenia and neutropenia were recorded as white blood cell count decreased and neutrophil count decreased, respectively

^b^TEAEs for the breast cancer subgroup were identified using the list of TEAEs occurring in >15% of patients in the safety population

*AEs* Adverse events, *CTCAE* Common Terminology Criteria for Adverse Events, *TEAEs* Treatment-emergent adverse events

Treatment-emergent paclitaxel-related or radium-223–related adverse events with a severity of grade ≥ 3 are shown in Supplementary Table S3; hematologic AEs were most commonly determined by the investigator as related to paclitaxel or radium-223 treatment.

A total of six patients experienced 11 SAEs: general physical health deterioration (grade 5 in 3 patients), fall (grade 3), hypophosphatemia (grade 3), tachycardia (grade 2), inguinal hernia (grade 3), pneumonia (grade 2 in 1 patient; grade 3 in 1 patient), femoral neck hip fracture (grade 5; details provided above), and hydronephrosis (grade 3). There were no deaths during treatment or within 30 days after permanent treatment discontinuation. The four patients with grade 5 AEs died >30 days after treatment discontinuation; the three cases of grade 5 general physical health deterioration were associated with clinical disease progression. Details on the patient with grade 5 hip fracture are discussed above; this patient also had grade 3 pneumonia classified as an SAE which the investigator considered related to paclitaxel and to radium-223 treatment. The grade 2 pneumonia SAE was considered related to paclitaxel, but not to radium-223 according to the investigator. All other SAEs were considered by the investigators as not related to either paclitaxel or radium-223 treatment. During the study, there were no episodes of bleeding or hemorrhage (any CTCAE grade) and no development of any new primary malignancy.

## Discussion

This open-label, multicenter, nonrandomized phase Ib study evaluated the safety of concomitant treatment with radium-223, a targeted alpha therapy, and paclitaxel, a taxane chemotherapy, in cancer patients with bone metastases. The primary end point was percentage of patients with grade 3/4 neutropenia or thrombocytopenia during treatment with radium-223 plus paclitaxel (cycles 2 and 3) versus paclitaxel alone (cycle 1). Paclitaxel was given alone during the first cycle to establish a baseline comparator for hematologic values observed in cycles 2 onward when radium-223 was given in combination with paclitaxel. Due to the high variability of hematologic changes among patients, a study design with an intra-individual comparison allowed for the observation of the hematologic effect of paclitaxel alone versus radium-223 in combination with paclitaxel.

In the 13 patients from the safety population who completed cycles 1–3, grade 3 neutropenia rates were 31% in cycle 2 versus 23% in cycle 1, and 8% in cycle 3 versus 23% in cycle 1; there were no cases of grade 4 neutropenia or grade 3/4 thrombocytopenia during cycles 1–3. When compared with neutropenia rates in cycle 1 (paclitaxel alone), results showed that combining treatment with radium-223 and paclitaxel (cycle 2) resulted in an increase in grade 3 neutropenia rates; however, the combination did not seem to increase rates of high-grade thrombocytopenia. The lower grade 3 neutropenia rate observed in cycle 3 versus cycle 2 was likely driven by paclitaxel dose modifications (either dose interruptions or dose reductions) or G-CSF use; however, no patients discontinued treatment due to toxicity from the treatment combination.

Safety results from the breast cancer subgroup were generally consistent with those of the safety population. When compared with the safety population, the breast cancer subgroup had slightly higher hematologic AE rates, but fewer grade 3/4 and serious TEAEs; more breast cancer patients also completed study treatment. The use of radium-223 in patients with breast cancer is currently being evaluated in phase II trials (i.e., NCT02258464, NCT02258451, and NCT02366130).

This study was the first to evaluate concomitant use of radium-223 and paclitaxel, and it was also the first to enroll patients with solid tumors not previously studied with radium-223 (i.e., bladder, non–small cell lung, myxofibrosarcoma, and neuroendocrine). Study limitations included the small sample size and the lack of formal or consistent collection of efficacy data. The small sample size may not have permitted the identification of AEs or SAEs that could occur with the combination. Additional study limitations were that patients with compromised bone marrow function were excluded, as were patients with an ECOG performance status >1. Additionally, while it is interesting to compare the breast cancer subgroup with the safety population, these results should be viewed as exploratory and hypothesis-generating given the small patient numbers. Larger, prospective trials are required to further evaluate the combination of radium-223 and paclitaxel in cancer patients with bone metastases.

Cancer treatment strategies are increasingly using combination therapies to treat the disease. Successful combinations often stem from agents with complementary mechanisms of action and non-overlapping toxicity profiles. In view of the known potential for myelosuppression during administration of radium-223 and paclitaxel as single agents [11, 16], it was clinically relevant to assess the potential for additive hematologic toxicity during coadministration of the drugs. Since patients with bone metastases are prone to increased myelosuppression with systemic chemotherapy, paclitaxel was chosen to be administered in combination with radium-223 in this study because it has a generally moderate effect on neutrophil counts, particularly in patients who have been heavily pretreated [14]. Additionally, paclitaxel is in the same drug class as docetaxel [14], so administering paclitaxel in combination with radium-223 was expected to have a similar tolerability profile as the randomized phase I/IIa radium-223 plus docetaxel study while generating data for potential new combination treatment strategies [13]. The paclitaxel dosing schedule was selected based on studies showing less hematologic toxicity when paclitaxel was administered in weekly versus every-3-week dosing [14]. Safety and tolerability findings from this study contribute to knowledge of the safety of radium-223 combined with taxane chemotherapy [13] and form the basis for the potential evaluation of radium-223 in other tumor types.

In conclusion, radium-223 was well tolerated when combined with paclitaxel in cancer patients with bone metastases, with no clinically relevant additive toxicities. In this study, combining treatment with radium-223 and paclitaxel resulted in an increase in neutropenia rates; however, thrombocytopenia rates did not appear to be impacted. A separate publication reporting the modeling data and potential mode of interaction between radium-223 and paclitaxel (whether additive or synergistic) is under preparation. The combination of radium-223 and paclitaxel should be explored further in cancer patients with bone metastases.

## Electronic supplementary material

ESM 1 (DOCX 33 kb)
