# Supplementary material for: Radium-223 in combination with paclitaxel in cancer patients with bone metastases: safety results from an open-label, multicenter phase Ib study
Source: Eur J Nucl Med Mol Imaging. 2018 Dec 13;46(5):1092–101. doi: 10.1007/s00259-018-4234-6 (PMC6451720; doi:10.1007/s00259-018-4234-6)
Supplement: Supplementary file 1 — (DOCX 33 kb) [file 259_2018_4234_MOESM1_ESM.docx]

**SUPPLEMENTARY DATA**

**Supplementary Table S1** Patient disposition

| Patient disposition | Total population  *N* = 22 | Breast cancer subgroup  *n* = 7 |
| --- | --- | --- |
| Enrolled, *n* (%) | 22 (100) | 7 (100) |
| Discontinued at screening, *n* (%) | 7 (32) | 0 |
| Screen failure | 5 (23) | NA |
| Patient withdrawal | 2 (9) | NA |
| Started treatment, *n* (%) | 15 (68) | 7 (100) |
| Discontinued treatment, *n* (%) | 8 (36) | 3 (43) |
| *Primary reason^a^* |  |  |
| AE associated with clinical disease progression | 1 (7) | 1 (14) |
| AE not associated with clinical disease progression | 2 (13) | 0 |
| Progressive disease—radiologic progression | 4 (27) | 1 (14) |
| Patient withdrawal | 1 (7) | 1 (14) |

^a^Percentages based on total number of patients treated (*n* = 15) and breast cancer patients (*n* = 7)

*AE* Adverse event, *NA* Not applicable

**Supplementary Table S2** Grade 3 or 4 neutropenia or thrombocytopenia in cycles 1-7

| Patients, *n* (%) | *n* | Neutropenia | | Thrombocytopenia | |
| --- | --- | --- | --- | --- | --- |
|  |  | Grade 3 | Grade 4 | Grade 3 | Grade 4 |
| Safety population | | | | | |
| Cycle 1 | 15 | 3 (20) | 1 (7) | 0 | 0 |
| Cycle 2 | 14 | 4 (29) | 1 (7) | 1 (7) | 0 |
| Cycle 3 | 13 | 1 (8) | 0 | 0 | 0 |
| Cycle 4 | 12 | 2 (17) | 0 | 0 | 0 |
| Cycle 5 | 9 | 1 (11) | 1 (11) | 0 | 0 |
| Cycle 6 | 8 | 1 (13) | 1 (13) | 0 | 0 |
| Cycle 7 | 7 | 0 | 0 | 0 | 0 |
| Breast cancer subgroup | | | | | |
| Cycle 1 | 7 | 2 (29) | 0 | 0 | 0 |
| Cycle 2 | 7 | 3 (43) | 0 | 0 | 0 |
| Cycle 3 | 7 | 1 (14) | 0 | 0 | 0 |
| Cycle 4 | 6 | 1 (17) | 0 | 0 | 0 |
| Cycle 5 | 5 | 0 | 1 (20) | 0 | 0 |
| Cycle 6 | 5 | 0 | 1 (20) | 0 | 0 |
| Cycle 7 | 4 | 0 | 0 | 0 | 0 |

**Supplementary Table S3** Treatment-emergent paclitaxel–related or radium-223–related adverse events with a severity of grade ≥3, as determined by the investigator

| Patients with TEAEs, n (%)^a^ | Safety population  *n* = 15 | Breast cancer subgroup  *n* = 7 |
| --- | --- | --- |
| Paclitaxel–related TEAEs of grade ≥3^b^ |  |  |
| Anemia | 1 (7) | 1 (14) |
| Leukopenia | 1 (7) | 0 |
| Neutropenia | 6 (40) | 3 (43) |
| Thrombocytopenia | 1 (7) | 0 |
| Fatigue | 1 (7) | 0 |
| Pneumonia | 1 (7) | 0 |
| Alanine aminotransferase increased | 1 (7) | 1 (14) |
| Lymphocyte count decreased | 1 (7) | 0 |
| White blood cell count decreased | 2 (13) | 2 (29) |
| Pain in extremity | 1 (7) | 0 |
| Rash erythematous | 1 (7) | 0 |
| Radium-223–related TEAEs of grade ≥3^b^ |  |  |
| Neutropenia | 3 (20) | 1 (14) |
| Thrombocytopenia | 1 (7) | 0 |
| Pneumonia | 1 (7) | 0 |
| Alanine aminotransferase increased | 1 (7) | 1 (14) |
| White blood cell count decreased | 1 (7) | 0 |
| Bone pain | 1 (7) | 0 |

^a^According to Medical Dictionary for Regulatory Activities (MedDRA) preferred term. Breast cancer subgroup according to CTCAE terminology: neutropenia was recorded as neutrophil count decreased.

^b^As determined by the investigator

*CTCAE* Common Terminology Criteria for Adverse Events, *TEAEs* Treatment-emergent adverse events
